# Monitoring and Risk Assessment of Multi-Pesticide Residues in Apples: A Focus on Consumer Safety

**DOI:** 10.3390/foods13193186

**Published:** 2024-10-07

**Authors:** Eylem Odabas, Mehmet Keklik, Ozgur Golge, Miguel Ángel González-Curbelo, Bulent Kabak

**Affiliations:** 1 Department of Food Engineering, Faculty of Engineering, Hitit University, Corum 19030, Turkey; eylem.odabas@ogrenci.hitit.edu.tr; 2 Air Alaşehir Food Control Laboratory, Alaşehir 45600, Turkey; 3 Department of Gastronomy and Culinary Arts, Faculty of Tourism, Alanya Alaaddin Keykubat University, Alanya 07425, Turkey; ozgur.golge@alanya.edu.tr; 4 Departamento de Ciencias Básicas, Facultad de Ingeniería, Universidad EAN, Calle 79 n° 11–45, Bogotá 110221, Colombia; magonzalez@universidadean.edu.co; 5 Biotechnology Laboratory, Machinery and Manufacturing Technology Application and Research Center, Hitit University, Corum 19030, Turkey

**Keywords:** food safety, dietary exposure, food contaminants, residue monitoring, sample preparation, method validation

## Abstract

Pesticide residues in human diets pose significant health hazards, particularly for vulnerable populations such as infants and children. This study aimed to determine pesticide residues in apples and to assess the cumulatively chronic risk posed to adult and child consumers from simultaneous exposure to multiple residues. During the 2022–2023 harvest seasons, 100 apple samples from Turkey were analyzed for the presence of 225 different pesticide residues. Pesticide extraction was performed using the QuEChERS method, followed by detection through liquid chromatography coupled with tandem mass spectrometry. Fifteen distinct pesticides (ten insecticides and five fungicides) were detected in 64 out of the 100 apple samples analyzed. Eleven samples contained pesticide residues that exceeded the maximum residue limit (MRL) set by the Turkish Food Codex and the European Union. Thiophanate-methyl was the most frequently detected pesticide (34%) in apples, with concentrations ranging from 0.012 to 0.108 mg kg^−1^, all of which were well below the MRL of 0.5 mg kg^−1^. Other commonly detected residues included chlorantraniliprole (28%), acetamiprid (24%), sulfoxaflor (22%), bifenazate (18%), indoxacarb (13%), diflubenzuron (12%), and carbendazim (10%). Under a worst-case scenario, the hazard index (HI) values for adults and children were 0.85% and 2.60%, respectively, indicating that these values remain significantly below the risk threshold of 100%, suggesting no associated health risks from apple consumption. However, regular monitoring of pesticide residues in fresh fruits and vegetables remains critically important.

## 1. Introduction

The global production and consumption of fruits are steadily increasing due to the growing adoption of healthy eating habits. The apple (*Malus domestica*) is a popular pome fruit worldwide because of its delicious taste and abundance of micronutrients and bioactive compounds. The nutritional composition of a medium-sized apple includes a significant amount of dietary fiber (18% of the recommended daily value), minerals (potassium, iron), vitamins (vitamin C, vitamin K, vitamin B6, riboflavin), and a variety of antioxidant components [1]. Apples’ nutritional value and health benefits are attributed to the presence of polyphenols (procyanidins, hydroxycinnamic acids, catechins, epicatechins, and quercetin) and other phytochemicals [1,2]. Apples are recognized for their antioxidant properties, which contribute to promoting heart health, reducing cancer risk and cardiovascular diseases, and enhancing the immune system [3]. In addition to fresh consumption, apples are widely used in apple juice, apple sauce, apple cider vinegar, various sweet and baked goods, and solid apple products for infants and young children.

Apple trees can grow in cold and temperate climates, with their origins traced back to the South Caucasus, including Anatolia, and their culture dates back to the B.C. era. The apple’s adaptation to many ecosystems has led to its widespread distribution [4]. In 2022, the global apple production area reached 4,825,729 hectares. According to the Food and Agriculture Organization (FAO) 2022 data, apples are the third most cultivated fruit in the world, following bananas (135 million metric tons) and watermelons (100 million metric tons), with a production amount of more than 95 million metric tons. In 2022, China was the world leader with approximately 45 million metric tons of apple production. Although the position of other nations in the ranking varies from year to year, Turkey ranks second in apple production, with a total of 4,817,500 metric tons in 2022, accounting for 5% of global output, followed by the USA, India, Russia, Italy, Iran, France, Chile, and Uzbekistan [5].

Several pests and diseases impact apple cultivation, reducing yield and fruit quality. Major diseases include fire blight (*Erwinia amylovora*), apple scab (*Venturia inaequalis*), Alternaria fruit rot (*Alternaria alternata*), brown rot (*Monilinia fructigena)*, and powdery mildew (*Podosphaera leucotricha*). In addition, pests such as codling moth (*Cydia pomonella*), woolly apple aphid (*Eriosoma lanigerum*), and San Jose scale (*Quadraspidiotus perniciosus*) can damage both trees and fruits, exacerbating losses [6].

Pest infestations represent a major threat to agricultural productivity, resulting in severe crop yield losses. The FAO reports that plant pests and diseases account for a 20–40% decrease in global crop yields each year. The increase in population and the effects of climate change exacerbate the situation, intensifying food insecurity and magnifying the losses. Moreover, climate change and variability are increasingly impacting agricultural yields, potentially exacerbating pest-related issues [7].

The use of pesticides plays a crucial role in increasing agricultural productivity, reducing crop losses, and preventing epidemic diseases. However, improper and excessive use of pesticides can lead to direct or indirect environmental and human health problems. Pesticides, while primarily applied to trees or soil, can also become airborne particles. These particles can be transported by wind or other atmospheric movements, reaching non-target plants, water bodies, and habitats, thereby exerting toxic effects on other living organisms [8]. Pesticides are a major cause of soil pollution and pose a significant threat to soil microorganisms, which contribute to the nutrient cycle and plant function. While soil microorganisms generally exhibit resistance to repeated pesticide applications, some species may be affected, leading to a decrease in microbial diversity. Furthermore, the amount of pesticide used can alter microorganisms’ metabolic rates and pathways. In the long term, the presence and persistence of pesticides can limit plants’ ability to grow in the soil, reducing the land’s suitability for agricultural and ecological purposes [9].

The use of pesticides is prevalent both domestically and internationally. Despite the availability of detailed usage instructions for licensed plant production products, it is observed that the majority of agricultural producers do not comply with these guidelines. This non-compliance not only impedes access to safe food for Turkish consumers but also causes issues at customs due to residue problems in exported products. The data for the year 2023 obtained from the Rapid Alert System for Food and Feed (RASFF) indicate that out of a total of 358 notifications originating from Turkey, the most prevalent hazard was pesticides, with a total of 167 notifications.

Therefore, monitoring and managing pesticide residues is critical for food safety. The European Union (EU) and other international organizations have established standards on this issue by setting maximum residue limits (MRLs) for pesticide residues in food products [10]. In Turkey, the Ministry of Agriculture and Forestry regulates and controls MRLs for pesticide residues in both animal and vegetable products [11].

As a result, monitoring pesticide residues in apples is of significant importance for both producers and consumers. While this monitoring helps produce healthy and safe food, it also contributes to environmental sustainability. Long-term and systematic monitoring programs allow pesticide use to be controlled and safer agricultural practices to be adopted. A range of monitoring programs have been implemented by relevant authorities and government institutions to oversee and monitor pesticide residues in different foodstuffs, with a particular focus on fruits and vegetables. However, a limited number of studies [12,13,14,15] have been conducted to ascertain the quantity of pesticide residues in apples and their potential health risks.

The main aims of this study were (i) to determine the presence of pesticide residues in apples consumed in Turkey, (ii) to compare the residue levels with the MRL values set by the EU, and (iii) to conduct a risk assessment based on apple consumption among adults and young children in Turkey. Between 2022 and 2023, 225 pesticide residues were monitored in 100 apple samples collected from various greengrocers, markets, and bazaars. Pesticide analysis in the apples was performed using the QuEChERS method followed by liquid chromatography-tandem mass spectrometry (LC-MS/MS). This study uniquely offers a comprehensive pesticide risk assessment for apple consumers in Turkey, focusing on cumulative exposure to multiple residues across different age groups. The data obtained will provide important information about the type and amount of pesticides used in apple production and help raise food safety standards by expanding the existing knowledge on the monitoring and evaluation of pesticide residues in apple production in Turkey.

## 2. Materials and Methods

### 2.1. Standards, Reagents and Chemicals

LC-MS-grade acetonitrile (C_2_H_3_N), methanol (CH_3_OH), and glacial acetic acid (CH_3_COOH, 100% purity) were supplied by Sigma-Aldrich (St. Louis, MO, USA). Analytical-grade anhydrous magnesium sulfate (MgSO_4_), anhydrous sodium acetate (C_2_H_3_NaO_2_), and LC-MS-grade formic acid (CH_2_O_2_, 98–100%) were procured from Merck (Darmstadt, Germany). Analytical-grade ammonium formate (HCOONH_4_, (≥99% purity)) was acquired from Sigma-Aldrich (Merck KGaA, Darmstadt, Germany). Primary–secondary amine (PSA) was purchased from Supelco^®^ (Bellefonte, PA, USA). Ultrapure water of 18.2 MΩ was provided by a Milli Q water purification system (Millipore, Molsheim, France).

The standards for 225 pesticides, belonging to different chemical families, were obtained from Dr. Ehrenstorfer GmbH (Augsburg, Germany), Sigma-Aldrich (Steinheim, Germany), and ChemService (West Chester, PA, USA), each with a purity greater than 95%. The selection included a range of pesticides commonly used in apple orchards, along with other active compounds, regardless of their registration status. An intermediate standard mix composed of 225 pesticides at a concentration of 10 µg mL^−1^ was prepared in acetonitrile. These standard mixtures were then utilized for spiking in recovery experiments and for creating matrix-matched calibration standards.

### 2.2. Samples

During the 2022–2023 harvest seasons, a total of 100 apple samples were analyzed for 225 pesticide residues. The apple samples were sourced from three distinct regions in Turkey: Isparta, Afyon, and Corum. The samples (1 kg), consisting of Starking and Golden varieties, were randomly collected at various greengrocers, markets, and bazaars located in Corum, Turkey. The apples, without any washing or other pre-treatment, were homogenized using a kitchen blender (Sefa Çelik, Beyoğlu, Istanbul, Turkey) before analysis.

### 2.3. Sample Preparation

**Figure 1:**
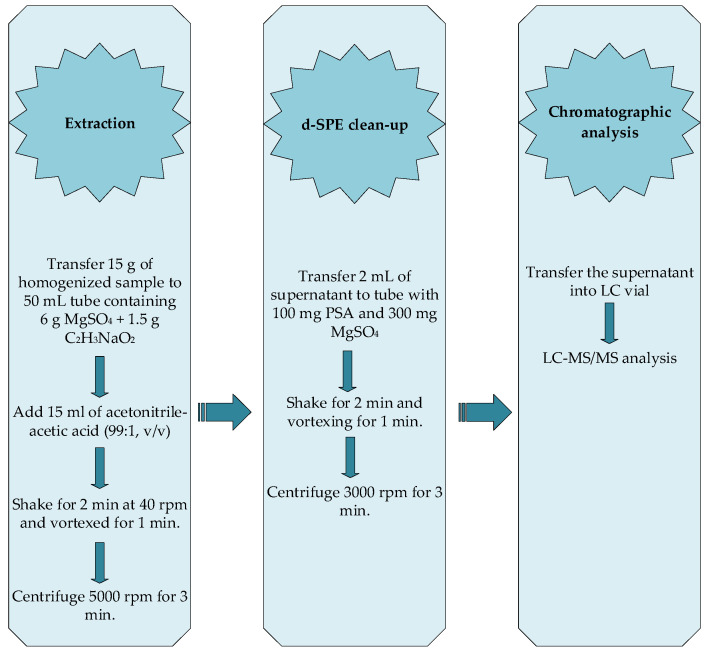
The procedure of the QuEChERS sample preparation method.

The QuEChERS extraction method, as proposed by the Association of Official Analytical Chemists (AOAC) [16], was used for the analysis of multiple residues in apple samples (Figure 1). The approach consists of two stages: liquid micro-extraction and the dispersive solid-phase extraction (d-SPE) cleanup step. Briefly, homogenized apple samples (15 g) were transferred into 50 mL capped centrifuge tubes containing 6 g of anhydrous MgSO_4_ and 1.5 g of C_2_H_3_NaO_2_. Subsequently, 15 mL of acetonitrile:acetic acid (99:1, *v*/*v*) extraction solvent was added to the tubes. The mixture was shaken for 2 min using a Multi RS-60 shaker (Biosan Ltd., Riga, Latvia) at 40 rpm and then vortexed for 1 min (Heidolph, Germany). This ensured thorough interaction between the solvent and the sample, effectively breaking down crystalline aggregates during shaking. The tubes were then centrifuged at 5000 rpm for 3 min to separate the phases. Two ml of supernatant was transferred to 15 mL centrifuge tubes containing 300 mg of anhydrous MgSO_4_ and 100 mg of PSA. The tubes were tightly capped and mixed on a shaker for 2 min, followed by vortexing for 1 min. The tubes were then centrifuged at 3000 rpm for 3 min. After centrifugation, 1 mL of the supernatant was transferred to a vial for further analysis.

### 2.4. LC-MS/MS Analysis

The detection and quantification of 225 pesticide residues in apple samples was performed using a Shimadzu LC-MS 8030 triple quadrupole mass spectrometer, which was integrated with a Shimadzu LC-20 series HPLC system (Tokyo, Japan). The multi-residue compounds were separated using an SVEA Core C-18 reversed-phase column (100 mm × 2.1 mm i.d., 2.6 µm particles) from Nanologica AB, Södertälje, Sweden. The column temperature was kept at 40 °C. The mobile phase was composed of eluant A (H_2_O-CH_3_OH, 98:2, *v*/*v*) and eluant B (CH_3_OH-H_2_O, 98:2, *v*/*v*), both solutions containing a mixture of 5 mM HCOONH_4_ and 0.1% CH_2_O_2_, with a flow rate of 0.6 mL min^−1^. The gradient was 80% A for 0.2 min, then ramped to 70% B from 0.2 min to 1.5 min, and then further ramped to 95% B by 5.0 min, followed by a 4 min hold time, after which the gradient was returned to 20% B by 9.1 min with a total run time of 10.0 min. The injection volume was 10 µL.

Pesticide residues were determined using multiple reaction monitoring (MRM) in either positive or negative ionization modes (ESI). Two MRM transitions were monitored for each residue following the SANTE/11312/2021 criteria [17]. The spray voltage was set to 4.5 kV. Nitrogen served as both the nebulizing and drying gases, with flow rates of 3.04 mL/min and 11 L/min, respectively. The heat block temperature was set at 400 °C, and the desolvation line temperature was maintained at 250 °C. The instrument control and data gathering were carried out using LabSolutions software version 5.19.

### 2.5. Method Validation

The analytical method was validated in accordance with the guidelines outlined in SANTE/11312/2021. The method was evaluated in-house regarding the linearity, limits of quantification (LOQs), recovery, and precision (repeatability and reproducibility). For the assessment of method linearity, a pesticide-free matrix was spiked with target analytes at six concentrations (0.005, 0.01, 0.02, 0.04, 0.08, and 0.16 mg kg^−1^), and matrix-matched calibration curves were created. The coefficients of determination (*R*^2^) for each target analyte were calculated. The LOQs, accuracy, and precision parameters were assessed using recovery trials. Pesticide-free matrix samples were spiked with a pesticide mixture at two concentration levels, 0.01 and 0.05 mg kg^−1^. The spiked samples were then analyzed following the method protocol, and the target analytes were quantified using matrix-matched calibration curves. The LOQs were assessed as ten times the standard deviation (SD) of replicate analyses of a blank sample matrix fortified with pesticide mix at 0.01 mg kg^−1^. Repeatability (*n* = 5) and within-laboratory reproducibility (*n* = 15) were determined as the relative standard deviations (%RSDs) of five replicate measurements on the same day and three consecutive days, respectively.

### 2.6. Risk Assessment

An evaluation of dietary exposure to residues was carried out to determine the chronic health risks for adults and children (aged 3–10) in Turkey. The estimation of dietary exposure to residues in apples is calculated by multiplying residue levels by consumption data, as demonstrated in Equation (1).

(1) $$Dietary\;exposure=\frac{Concentration\;of\;residue\;\left(\frac{mg}{kg}\right)\times Apple\;consumption\;(kg)}{Body\;weight\;(kg)}$$

The consumption trends for the Turkish population were obtained from the food balance sheet provided by the Turkish Statistical Institute [18]. This sheet revealed that the average annual consumption of apples is 29.5 kg per person, which is equivalent to 0.081 kg per capita per day. The assessment took into account both adult and child consumer groups, following the European Food Safety Authority (EFSA) guidelines [19], which recommend body weights of 70 kg for adults and 23 kg for children aged 3–10 years.

Throughout the exposure assessment process, there are unavoidable uncertainties in estimating dietary exposure to chemical hazards, including pesticides. These uncertainties stem from variations in food intake rates, individual body weights, sampling methods, analytical biases and fluctuations, processing factors, and left-censored data [20]. Handling concentration data that fall below the limit of detection (LOD) or LOQ, termed left-censored data, significantly contributes to uncertainty. The substitution method, as outlined in the EFSA scientific report [21], has been used for such data, in which non-detectable results are replaced with zero or the LOQ value, representing Lower (LB) and Upper Bound (UB) scenarios, respectively. The study did not consider additional sources of uncertainty.

The cumulative risk of pesticide exposure was assessed using the hazard index (*HI*) method, which involves summing the hazard quotients (*HQ*) of individual pesticides, as described by Reffstrup et al. [22]. The calculation of the *HQ* for a particular pesticide entails dividing the exposure to its residue by its acceptable daily intake (*ADI*). The calculations for *HQ* and *HI* are presented in Equations (2) and (3), respectively.

(2) $$Hazard\;Quotient\;\left(HQ\right)=\frac{Exposure\;of\;the\;concerned\;pesticide}{Reference\;value\;(ADI)}$$

(3) $$Hazard\;Index\;\left(HI\right)=\Sigma \;HQ$$

## 3. Results and Discussion

### 3.1. Validation Data

Before applying the LC-MS/MS method to apple samples, the effectiveness of the analytical method was evaluated using several criteria outlined in the SANTE/11312/2021 Guideline. The selectivity of the method was ensured through the use of MRM mode. Specifically, selectivity was validated by monitoring the transition ions, including one quantifier ion and two qualifier ions, at retention times corresponding to those of the target pesticide residues. The ratios of the peak areas for the qualifier to the quantifier ions were consistently within a relative deviation of ±30% from the average of the calibration standards run in the same sequence, as recommended by the guidelines outlined in SANTE 11312/2021. The linear matrix-matched calibration curves, constructed using six different concentration levels (0.005–0.16 mg kg^−1^), exhibited *R*^2^ greater than 0.99 for all compounds (Table S1). The LOQ values and the recovery and precision data for the monitored pesticide residues at two different concentrations (0.01 and 0.05 mg kg^−1^) are presented in Table S2. Acceptable linearity was obtained for all target analytes in the matrix. The LOQ values for the pesticide residues were ≤0.01 mg kg^−1^. The method demonstrated good recovery values for all compounds, with recoveries ranging from 74.3 to 115.3%. The method’s precision (0.7–19.8% RSD) was also found to be satisfactory for all compounds. Recoveries and precision values meet the performance values set by the SANTE/11312/2021 guideline.

### 3.2. Pesticides in Apples

Between 2022 and 2023, 100 apple samples collected from various greengrocers, markets, and bazaars in Turkey were monitored for 225 pesticide residues using an in-house validated LC-MS/MS method. The pesticides detected in the apple samples and their quantities are presented in Table 1.

Out of the 100 apple samples analyzed, 36 did not contain any measurable pesticide residues, whereas 53 samples had at least one detectable residue within the acceptable legal levels. In 11 apple samples, the detected residue levels for diflubenzuron and methoxyfenozide exceeded the EU MRL values.

**Figure 2:**
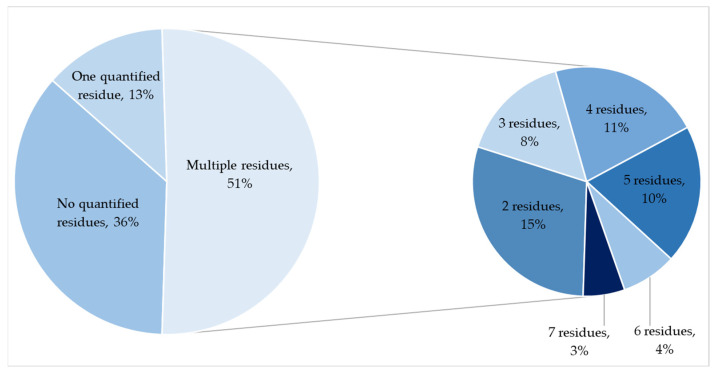
Number of quantified residues in apple samples.

In 13 apple samples, only one residue was identified, while 51 samples contained multiple residues (Figure 2). Among these 51 samples with multiple residues, 15 contained two pesticides, 8 contained three, 11 contained four, 10 contained five, 4 contained six, and 3 contained seven pesticide residues. As shown in Table 1, 15 different residues, including six unauthorized pesticides, were detected at quantifiable concentrations in the apple samples. Of the detected active substances, five were fungicides and ten were insecticides, two of which also exhibited acaricidal effects.

The insecticide thiophanate-methyl, which belongs to the benzimidazole group, was the most commonly found pesticide among the analyzed residues, with a detection frequency of 34%. Thiophanate-methyl was found in the apple samples at concentrations ranging from 0.012 to 0.108 mg kg^−1^, with an average of 0.040 mg kg^−1^. The high occurrence of thiophanate-methyl in apple samples is attributed to its application as a pre-harvest fungicide for managing certain mold infections, including *Monilinia laxa* and *Monilinia fructigena*, which are responsible for brown rot disease. Thiophanate-methyl is a broad-spectrum systemic fungicide with both protective and curative properties, absorbed by roots and leaves. The methyl benzimidazole carbamate family comprises systemic fungicides, such as benomyl, carbendazim, and thiophanate-methyl [23]. Thiophanate-methyl has been classified as a Category 2 mutagen and is being considered for classification as a Category 2 carcinogen based on dose-dependent increases in liver tumors in male and female mice [24,25]. The ADI for thiophanate-methyl is 0.02 mg kg^−1^ b.w. day^−1^, and the acute reference dose (ARfD) is 0.02 mg kg^−1^ [25]. The degradation of thiophanate-methyl into carbendazim, which is an intermediate metabolite, has been observed in apples, grapes, and beans. The main chemicals detected following the application of thiophanate-methyl to stone fruit leaves were the original compound and carbendazim. These compounds constituted 64.5% (thiophanate-methyl) and 22.2% (carbendazim) of the total radioactive residue (TRR) in 1-day samples, and 44.5% (thiophanate-methyl) and 33.4% (carbendazim) of the TRR in 7-day samples [26]. In this study, the detection rates of thiophanate-methyl and carbendazim were found to be 34% and 10% in apples, respectively. These results indicate that thiophanate-methyl and carbendazim, which are non-approved in the EU, are still used as plant protection products in Turkey.

Chlorantraniliprole was the second most commonly recorded residue in apple samples. This insecticide was found in 28 apple samples at concentrations ranging from 0.02 to 0.085 mg kg^−1^, with an average of 0.044 mg kg^−1^. The frequent presence of chlorantraniliprole in apple samples is attributed to its use as a pre-harvest insecticide against the codling moth (*Cydia pomenella*). Chlorantraniliprole, an anthranilic diamide insecticide, is effective against pests at all larval stages and some species’ eggs. It causes the depletion of calcium stores in the insect’s smooth and striated muscles, resulting in muscle weakness and death [27]. Chlorantraniliprole demonstrates low acute toxicity and does not display any genotoxic or carcinogenic properties. The ADI for chlorantraniliprole is set at 1.56 mg kg^−1^ b.w. day^−1^ [28].

Acetamiprid was the third most commonly quantified residue in apple samples, with a detection frequency of 24%. Acetamiprid was found in the apple samples at concentrations ranging from 0.011 to 0.054 mg kg^−1^, with an average of 0.031 mg kg^−1^. Acetamiprid, a neonicotinoid insecticide, is used to protect fruits and vegetables, including apples, from certain pests such as Hemiptera, Lepidoptera, Thysanoptera, and Coleoptera [29]. The prevalence of acetamiprid in apple samples is attributed to its use as a pre-harvest insecticide against the apple aphid (*Aphis pomi*). Acetamiprid exhibits relatively low acute and chronic toxicity in mammals with no evidence of carcinogenicity, neurotoxicity, mutagenicity, or endocrine-disrupting effects. The ADI for acetamiprid is 0.025 mg kg^−1^ b.w. day^−1^, and the ARfD is 0.25 mg kg^−1^ b.w. [30].

Other pesticides recorded in apple samples with a frequency of 10% or higher were sulfoxaflor (22%), bifenazate (18%), indoxacarb (13%), diflubenzuron (12%), and carbendazim (10%). The concentrations of sulfoxaflor, bifenazate, indoxacarb, and carbendazim in apple samples ranged from 0.013 to 0.045 mg kg^−1^ (mean = 0.043 mg kg^−1^), 0.011 to 0.052 mg kg^−1^ (0.029 mg kg^−1^), 0.013 to 0.072 mg kg^−1^ (0.030 mg kg^−1^), and 0.010 to 0.156 mg kg^−1^ (0.081 mg kg^−1^), respectively. In addition, the residues myclobutanil (9%, 0.016–0.035 mg kg^−1^), pyridaben (9%, 0.013–0.032 mg kg^−1^), spirodiclofen (9%, 0.012–0.029 mg kg^−1^), boscalid (8%, 0.014–0.038 mg kg^−1^), trifloxystrobin (4%, 0.012–0.029 mg kg^−1^), flubendiamide (3%, 0.017–0.033 mg kg^−1^), and methoxyfenozide (2%, 0.010–0.025 mg kg^−1^) were rarely detected in apple samples with a frequency below 10%.

The current findings are comparable to prior studies in which a limited number of samples and residues were monitored. In a previous study, the presence and quantities of 203 pesticide residues were monitored in 46 apple samples marketed in Konya, Turkey [12]. At least one pesticide was detected in 54.3% of the apple samples. The two most frequently found residues in apple samples were acetamiprid (26%) and carbendazim (13%). The levels of acetamiprid and carbendazim detected in samples ranged from 0.004 to 0.089 mg kg^−1^ and from 0.002 to 0.089 mg kg^−1^, respectively. These two pesticides’ detection frequencies are similar to those obtained from this study. In another study, the presence and quantities of 13 different residues from the organophosphate and pyrethroid groups were investigated in 35 apple samples from the 2008–2009 and 2009–2010 harvest years grown in Isparta province. Dichlorvos residues were observed in four apple samples from the 2008–2009 harvest year, ranging from 0.2 to 0.4 mg kg^−1^, and in two apple samples from the 2009–2010 crop year, at 0.2 and 0.3 mg kg^−1^ [13].

In a study conducted on the Granny Smith apple variety in the Gürsu district of Bursa, the residual amounts and half-lives between the last application and harvest were determined for pyridaben and tebuconazole under orchard conditions. The pesticide residues on apples harvested 1, 6, 14, and 21 days after application were found to be 0.575, 0.141, 0.137, and 0.075 mg kg^−1^ for pyridaben, and 0.418, 0.160, 0.108, and 0.046 mg kg^−1^ for tebuconazole, respectively. The researchers also concluded that a period of 21 days between the last spraying and harvest would be appropriate [14]. More recently, the concentrations of imidacloprid and indoxacarb residues in apples harvested 3 and 14 days after spraying in orchards located in Çanakkale were examined by Özel and Tiryaki [15]. The residue levels of imidacloprid and indoxacarb were 0.211 and 0.129 mg kg^−1^, and 0.564 and 0.418 mg kg^−1^, respectively.

Several studies have been carried out in various countries on the presence of pesticide residues in apples. A monitoring program conducted in Denmark from 2004 to 2011 found 12 different residues in 46% of domestically produced apple samples, with 2% exceeding the MRL. In imported samples, 80% contained 54 different residues, and 3% exceeded the MRL. The prevalence of multiple residues was 39% [31]. In a Polish survey from 2005–2013, 696 apple samples were monitored for 182 residues. A total of 33.5% of samples did not contain any residues, while 66.5% of samples contained 34 different residues at measurable concentrations. In 3% of the samples (21 samples), the residue concentrations exceeded the MRL. The most frequently recorded compounds were dithiocarbamates (21.4%), captan/folpet (19.3%), diphenylamine (14.6%), and chlorpyrifos (13.2%) [32].

In 2023 in China, Zhao et al. [33] analyzed 120 apple samples for the presence of 14 pesticide residues. Pesticides were detected in 91.7% of the samples, with the most frequent being thiamethoxam (63.3%), carbendazim (61.7%), pyraclostrobin (22.5%), tebuconazole (21.7%), chlorpyrifos (8.3%), and fenproxymate (2.5%). In Serbia, 34 apple samples were monitored for pesticide residues. A total of 23.5% of 34 samples (8 samples) were free from target residues, while 21 samples (61.8%) contained at least one residue but remained below the legal limits. In five samples, the concentrations of residues were higher than MRLs. Acetamiprid, captan, cypermethrin, fludioxonil, carbendazim, and chlorantraniliprole were reported to be the most frequently identified pesticides in apples from Serbia [34].

The 2013 official control activities of EU member states, Iceland, and Ireland provided the occurrence data of pesticide residues for a total of 1610 apple samples. Out of these samples, no residues were found in 533 samples (33%), a single residue was found in 338 samples (21.0%), and multiple residues were found in 739 samples (45.9%). Sixteen samples (1%) contained residues exceeding EU MRLs. Fifty-five different pesticides were detected, with the most frequent being captan/folpet (27.9%), dithianon (23%), and dithiocarbamates (17.7%) [35]. According to the EFSA’s 2015 report on pesticide residues in food, 898 out of 2867 apple samples (31.3%) were free of pesticides, 587 samples (20.5%) had only one residue, and 1382 samples (48.2%) contained multiple residues. Eleven samples contained more than ten different pesticides [36].

The results of this study highlight important considerations regarding regulatory actions related to detected pesticide residues. Notably, eleven apple samples exceeded the MRL, highlighting the need for enhanced regulatory oversight. In Turkey, where agricultural practices vary, these results suggest a potential gap in compliance and monitoring efforts. Considering that thiophanate-methyl is not authorized for use within the EU and Turkey, regulatory authorities in Turkey and the EU must assess the implications of its frequent detection, alongside other residues, on consumer health. Regulatory authorities must consider implementing more stringent testing protocols and increasing public awareness about pesticide exposure risks. Furthermore, the implications extend to EU policies, which may necessitate adjustments in monitoring frameworks to ensure consumer safety. Ongoing evaluation of pesticide regulations and their enforcement will be essential to mitigate health risks associated with cumulative pesticide exposure, particularly for vulnerable populations.

### 3.3. Risk Assessment

The analysis of apple samples revealed that for 210 out of the 225 monitored pesticide residues, no detectable residues were found. This suggests that exposure to these residues can be considered negligible. The chronic exposure levels and HQs of 15 different pesticides for adults and children are presented in Table 2.

For adults, the exposure levels to various pesticides through apple consumption ranged from 7.4 × 10^−7^ to 1.6 × 10^−5^ mg kg^−1^ b.w. day^−1^ at the LB and from 3.4 × 10^−6^ to 2.1 × 10^−5^ mg kg^−1^ b.w. day^−1^ at the UB. For children aged 3–10 years, the exposure levels ranged from 2.3 × 10^−6^ to 4.8 × 10^−5^ mg kg^−1^ b.w. day^−1^ at the LB, and from 1.0 × 10^−5^ to 6.5 × 10^−5^ mg kg^−1^ b.w. day^−1^ at the UB. Thiophanate-methyl, frequently detected in apple samples, exhibited the highest exposure levels for both adults and children. The cumulative exposure to pesticides through apple consumption for adults and children was 8.5 × 10^−5^ and 2.6 × 10^−4^ mg kg^−1^ b.w. day^−1^ at the LB and 1.7 × 10^−4^ and 5.3 × 10^−4^ mg kg^−1^ b.w. day^−1^ at the UB, respectively.

**Figure 3:**
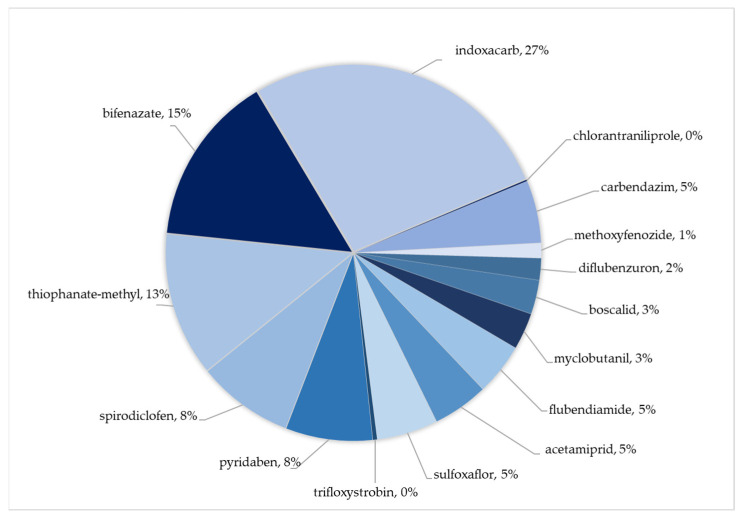
Contribution of detected residues to HI for apples.

The HQs of individual pesticide residues for adults ranged from 0.001% to 0.091% at the LB and from 0.001% to 0.232% at the UB. For children, the HQs ranged from 0.002% to 0.278% at the LB and from 0.004% to 0.707% at the UB. For both adults and children, the HQs of all pesticides were found to be below 1%. Compared to adults, children exhibited higher HQs due to their lower body weight. The contributions of detected pesticides to the HI for adults and children are shown in Figure 3.

Under the worst-case scenario, the HI value was 0.85% for adults and 2.60% for children, indicating that pesticide exposure through apple consumption does not pose a significant health risk for the Turkish population. While thiophanate-methyl had the highest contribution to cumulative exposure, indoxacarb contributed the most to HI for both adults and children (27%). Two other pesticides that contributed over 10% to the HI were bifenazate (15%) and thiophanate-methyl (13%).

A similar study by Poulsen et al. [31] in Denmark reported an exposure level of 5.7 × 10^−4^ mg kg^−1^ b.w. day^−1^ from apple consumption, aligning with the results of the present study. In that study, the HI for pesticide residues from apple consumption was 6.9%, which is 2.7–8 times higher than the HI observed in our study. In another study, the HQs for the individual pesticides varied from 0.01% to 75.8%. Most of the HQs were below 100%, signifying that the consumption of apples does not pose any risk of adverse effects from individual pesticides. The compound diazinon was found to have the highest contribution; however, it did not surpass 76% of the ADI intake for the most critical population of toddlers, specifically the United Kingdom toddler population [32].

## 4. Conclusions

This study aimed to evaluate pesticide residues in apples and assess the potential health risks associated with these residues for adults and children in Turkey. The analytical method, which involved QuEChERS sample preparation followed by LC-MS/MS analysis, was successfully validated for detecting and quantifying 225 residues in apples. Of the apple samples analyzed, 36 were found to contain no measurable pesticide residues, while 64 samples had residues of 15 different pesticides. In 11 of these samples, the residue levels for two pesticides (diflubenzuron and methoxyfenozide) exceeded the MRLs. Among the samples with detected residues, 13 contained a single residue, whereas 51 samples had multiple pesticide residues. Furthermore, six of the detected pesticide residues were not authorized for use in the EU. The most frequently detected pesticide was thiophanate-methyl (34%), followed by chlorantraniliprole (28%), acetamiprid (24%), sulfoxaflor (22%), bifenazate (18%), indoxacarb (13%), diflubenzuron (12%), and carbendazim (10%). The cumulative exposure values for pesticides through apple consumption ranged from 8.5 × 10^−5^ to 1.7 × 10^−4^ mg kg^−1^ b.w. day^−1^ for adults and from 2.6 × 10^−4^ to 5.3 × 10^−4^ mg kg^−1^ b.w. day^−1^ for children. The HI values, under worst-case scenarios, were 0.85% for adults and 2.60% for children, indicating that pesticide exposure through apple consumption is not a significant health concern. The pesticides contributing most to the hazard index were indoxacarb (27%), bifenazate (15%), and thiophanate-methyl (13%). These values are notably lower than established benchmarks in previous studies. This contrast underscores the effectiveness of current monitoring practices and agricultural regulations. This indicates improved pesticide management in apple cultivation, yet the presence of residues exceeding the MRL underscores the need for continued vigilance. By demonstrating comparatively lower exposure levels, this study not only highlights progress but also sets a foundation for future regulatory actions and consumer safety initiatives. Regular monitoring of residues in apples and other fruits and vegetables by the Food and Control General Directorate of the Ministry of Agriculture and Forestry is crucial to ensuring the safety of the food supply in Turkey. To mitigate the hazards associated with pesticide residues and minimize their adverse health effects, specific intervals between pesticide application and harvest should be established for each pesticide and crop, and producers should be educated on these intervals. Additionally, there should be a greater emphasis on advancing biological pest control methods (biopesticides) rather than relying solely on synthetic pesticides and fertilizers. To further reduce health risks from pesticide exposure, consumers should be educated on the importance of thoroughly washing fruits and vegetables under running water, as well as peeling them, since washing alone may not fully remove pesticide residues due to skin penetration.

## Figures and Tables

**Table 1:** The distribution and concentration of pesticides in apples.

Pesticide	Pesticide Type	EU MRL (mg kg^−1^)	% of Samples < LOQ	% of Samples LOQ-MRL	% of Samples > MRL	Range (mg kg^−1^)	
						Min.–Max.	Mean
Acetamiprid	IN ^a^	0.4	76	24	-	0.011–0.054	0.031
Bifenazate	IN	0.7	82	18	-	0.011–0.052	0.029
Boscalid	FU ^b^	2.0	92	8	-	0.014–0.038	0.027
Carbendazim *	FU	0.2	90	10	-	0.011–0.047	0.026
Chlorantraniliprole	IN	0.4	72	28	-	0.020–0.085	0.044
Diflubenzuron *	IN	0.01	88	1	11	0.010–0.156	0.081
Flubendiamide	IN	0.9	97	3	-	0.017–0.033	0.026
Indoxacarb *	IN	0.5	87	13	-	0.013–0.072	0.030
Methoxyfenozide	IN	0.01	96	2	2	0.010–0.025	0.016
Myclobutanil *	FU	0.6	91	9	-	0.016–0.035	0.024
Pyridaben	IN/AC ^c^	0.9	91	9	-	0.013–0.032	0.020
Spirodiclofen *	IN/AC	0.8	91	9	-	0.012–0.029	0.021
Sulfoxaflor	IN	0.4	78	22	-	0.013–0.045	0.043
Thiophanate-methyl *	FU	0.5	66	34	-	0.012–0.108	0.040
Trifloxystrobin	FU	0.7	96	4	-	0.010–0.045	0.025

^a^ IN: insecticide. ^b^ FU: fungicide. ^c^ AC: acaricide. * Not approved in the EU.

**Table 2:** Exposure and HQ for detected pesticides in apples for the consumer group of adults and children.

Pesticide	ADI (mg kg^−1^ b.w. day^−1^)	Adult		Children	
		Exposure (mg kg^−1^ b.w. day^−1^)	HQ	Exposure (mg kg^−1^ b.w. day^−1^)	HQ
Acetamiprid	0.025	8.5 × 10^−6^ (LB ^a^)	0.034	2.6 × 10^−5^ (LB)	0.104
		1.0 × 10^−5^ (UB ^b^)	0.041	3.1 × 10^−5^ (UB)	0.125
Bifenazate	0.01	6.0 × 10^−6^ (LB)	0.060	1.8 × 10^−5^ (LB)	0.183
		1.3 × 10^−5^ (UB)	0.126	3.9 × 10^−5^ (UB)	0.385
Boscalid	0.04	2.5 × 10^−6^ (LB)	0.006	7.6 × 10^−6^ (LB)	0.019
		9.9 × 10^−6^ (UB)	0.025	3.0 × 10^−5^ (UB)	0.076
Carbendazim *	0.02	3.0 × 10^−6^ (LB)	0.015	9.2 × 10^−6^ (LB)	0.046
		9.3 × 10^−6^ (UB)	0.046	2.8 × 10^−5^ (UB)	0.141
Chlorantraniliprole	1.56	1.4 × 10^−5^ (LB)	0.001	4.3 × 10^−5^ (LB)	0.003
		2.0 × 10^−5^ (UB)	0.001	6.1 × 10^−5^ (UB)	0.004
Diflubenzuron *	0.1	1.0 × 10^−5^ (LB)	0.010	3.1 × 10^−5^ (LB)	0.031
		1.6 × 10^−5^ (UB)	0.016	5.0 × 10^−5^ (UB)	0.050
Flubendiamide	0.017	9.0 × 10^−7^ (LB)	0.005	2.8 × 10^−6^ (LB)	0.016
		6.5 × 10^−6^ (UB)	0.038	2.0 × 10^−5^ (UB)	0.117
Indoxacarb *	0.005	4.6 × 10^−6^ (LB)	0.091	1.4 × 10^−5^ (LB)	0.278
		1.2 × 10^−5^ (UB)	0.232	3.5 × 10^−5^ (UB)	0.707
Methoxyfenozide	0.1	7.4 × 10^−7^ (LB)	0.001	2.3 × 10^−6^ (LB)	0.002
		1.1 × 10^−5^ (UB)	0.011	3.3 × 10^−5^ (UB)	0.033
Myclobutanil *	0.025	2.5 × 10^−6^ (LB)	0.010	7.7 × 10^−6^ (LB)	0.031
		6.7 × 10^−6^ (UB)	0.027	2.1 × 10^−5^ (UB)	0.082
Pyridaben	0.01	2.1 × 10^−6^ (LB)	0.021	6.5 × 10^−6^ (LB)	0.065
		6.4 × 10^−6^ (UB)	0.064	1.9 × 10^−5^ (UB)	0.193
Spirodiclofen *	0.015	2.2 × 10^−6^ (LB)	0.015	6.6 × 10^−6^ (LB)	0.044
		1.1 × 10^−5^ (UB)	0.071	3.2 × 10^−5^ (UB)	0.215
Sulfoxaflor	0.04	1.1 × 10^−5^ (LB)	0.027	3.3 × 10^−5^ (LB)	0.083
		1.8 × 10^−5^ (UB)	0.045	5.6 × 10^−5^ (UB)	0.138
Thiophanate-methyl *	0.02	1.6 × 10^−5^ (LB)	0.079	4.8 × 10^−5^ (LB)	0.241
		2.1 × 10^−5^ (UB)	0.106	6.5 × 10^−5^ (UB)	0.323
Trifloxystrobin	0.1	1.1 × 10^−6^ (LB)	0.001	3.5 × 10^−6^ (LB)	0.003
		3.4 × 10^−6^ (UB)	0.003	1.0 × 10^−5^ (UB)	0.010

^a^ LB: lower bound (results below the LOQ were replaced with 0); ^b^ UB: upper bound (results below the LOQ were replaced with the value of LOQ). * Not approved in the EU.

## Data Availability

The original contributions presented in the study are included in the article and Supplementary Material, further inquiries can be directed to the corresponding author.

## Supplementary Materials

The following supporting information can be downloaded at: https://www.mdpi.com/article/10.3390/foods13193186/s1, Table S1: Linear equation of calibration curve for the quantification of the 15 pesticide residues; Table S2: In-house validation data for 225 residues by LC-MS/MS.
