# Supplementary material for: Monitoring and Risk Assessment of Multi-Pesticide Residues in Apples: A Focus on Consumer Safety
Source: Foods. 2024 Oct 7;13(19):3186. doi: 10.3390/foods13193186 (PMC11476224; doi:10.3390/foods13193186)
Supplement: Supplementary file 1 [file foods-13-03186-s001.zip › foods-3229991-supplementary.pdf]

Supplementary material

Table S1. Linear equation of calibration curve for the quantification of the 15 pesticide residues.

**Pesticide**	**Linear equation**	$\boldsymbol{R^2}$
Acetamiprid	$y = 145019x + 13705$	0.9996
Bifenazate	$y = 30333x + 98222$	0.9990
Boscalid	$y = 56256x + 30593$	0.9993
Carbendazim	$y = 92189x + 26724$	0.9994
Chlorantraniliprole	$y = 632456x + 40404$	0.9984
Diflubenzuron	$y = 30060x + 11252$	0.9955
Flubendiamide	$y = 30230x + 15129$	0.9978
Indoxacarb	$y = 37260x + 60720$	0.9988
Methoxyfenozide	$y = 43367x + 17165$	0.9988
Myclobutanil	$y = 60650x + 39621$	0.9977
Pyridaben	$y = 91300x + 40211$	0.9993
Spirodiclofen	$y = 20524x + 27640$	0.9995
Sulfoxaflor	$y = 35144x + 16672$	0.9985
Thiophanate-methyl	$y = 42501x + 30357$	0.9969
Trifloxystrobin	$y = 36964x + 11017$	0.9992

R^2: coefficient of determination

Table S2. In-house validation data for 225 residues by LC-MS/MS.

Pesticide	LOQ (mg kg⁻¹)	Recovery (%)		Repeatability (%RSD$_r$, n=5)		Within-laboratory reproducibility (%RSD$_R$, n=15)	
		0.01 mg kg^{-1}	0.05 mg kg^{-1}	0.01 mg kg^{-1}	0.05 mg kg^{-1}	0.01 mg kg^{-1}	0.05 mg kg^{-1}
Acetamiprid	0.002	79.5	91.1	8.3	4.0	10.9	6.9
Aldicarb	0.007	77.6	89.5	6.7	3.1	8.4	5.8
Aldicarb-sulfone	0.006	76.7	98.1	2.2	5.5	19.3	3.3
Aldicarb-sulfoxide	0.006	82.8	81.9	3.0	6.0	4.1	7.6
Aminocarb	0.007	77.1	85.7	15.2	2.9	17.1	5.3
Amitraz	0.003	84.0	86.0	4.9	5.9	15.9	11.5
Azinphos-ethyl	0.006	91.1	112.7	9.1	1.4	9.7	2.9
Azinphos-methyl	0.005	83.7	98.0	9.2	0.7	10.3	5.4
Azoxystrobin	0.003	77.8	94.2	11.2	5.7	14.0	10.3
Benalaxyl	0.002	80.7	93.3	2.3	3.9	18.2	9.4
Bendiocarb	0.005	104.0	94.6	13.3	5.4	9.4	8.4
Bifenazate	0.007	89.5	96.7	9.7	3.5	7.1	6.7
Bitertanol	0.008	76.2	87.5	8.7	3.1	5.1	3.0
Boscalid	0.007	79.4	88.0	13.9	5.9	4.8	9.6
Bromoxynil	0.006	78.3	82.1	7.7	5.7	9.2	4.6
Bromuconazole	0.007	103.4	100.8	9.8	8.1	11.3	8.8
Bupirimate	0.002	79.5	95.2	12.0	6.2	6.7	4.9
Buprofezin	0.002	79.4	90.7	18.1	4.7	4.6	13.3
Butachlor	0.003	87.5	102.1	11.4	8.9	7.5	6.7
Butafenacil	0.007	91.5	92.4	10.6	3.0	14.3	5.9
Buturon	0.003	87.9	100.8	7.6	3.6	18.5	14.8
Butylate	0.005	80.9	97.3	13.6	3.3	18.6	8.2
Cadusafos	0.002	87.7	99.1	3.7	4.1	15.3	9.6
Carbaryl	0.003	83.1	92.9	15.6	7.5	9.5	9.0
Carbendazim	0.006	98.4	85.1	4.4	2.7	6.5	8.2
Carbofuran	0.005	88.2	102.1	9.9	2.5	18.5	6.6
Carboxin	0.003	78.0	90.7	12.5	3.6	12.8	8.3
Chlorantraniliprole	0.007	89.5	92.3	16.3	5.5	12.0	7.2
Chlorbromuron	0.007	86.8	91.1	13.6	7.4	17.5	8.7

Chlorfluazuron	0.006	78.8	77.7	7.3	4.7	8.3	3.8
Chloridazon	0.003	78.9	90.0	7.6	2.3	4.4	7.9
Chlorpyrifos	0.004	80.8	91.2	6.7	0.7	10.9	3.2
Chlorpyrifos-methyl	0.004	83.5	89.2	7.4	2.0	8.3	5.0
Chlorsulfuron	0.002	81.5	81.2	2.2	5.8	17.5	10.1
Clethodim	0.003	83.2	92.6	11.7	2.4	13.7	10.3
Clofentezine	0.010	101.1	94.8	6.7	4.5	4.7	7.0
Clomazone	0.002	92.9	107.8	7.5	4.3	18.7	10.2
Clothianidin	0.009	76.6	89.7	7.7	1.8	17.9	3.2
Cyclohexamide	0.008	80.7	82.1	13.4	8.5	10.8	6.3
Cyflufenamid	0.003	96.4	112.4	15.4	11.6	10.7	10.4
Cymoxanil	0.007	82.7	84.7	9.4	3.4	5.2	5.5
Cyproconazole	0.008	81.1	91.9	14.2	2.5	11.4	8.8
Cyprodinil	0.003	80.9	95.2	4.9	4.8	8.8	8.5
Deltamethrin	0.009	105.4	111.7	4.7	3.1	7.4	11.3
Demeton-s (disulfoton oxon)	0.005	90.6	112.3	7.2	6.0	6.0	7.3
Demeton-s-methyl-sulfone	0.008	77.4	83.7	11.2	4.2	10.8	5.5
Demeton-s-methyl-sulfoxide	0.008	77.2	82.2	16.3	4.1	10.9	9.8
Desmedipham	0.002	77.1	93.2	9.9	3.0	13.9	5.3
Desmetryn	0.002	82.2	90.2	16.9	2.6	14.6	3.4
Diafenthiuron	0.005	84.6	99.3	8.4	3.6	14.3	9.5
Diazinon	0.003	82.9	98.9	4.2	4.4	18.4	4.7
Dichlorvos	0.007	81.5	94.7	12.0	2.9	14.7	6.2
Diclobutrazol	0.006	78.6	92.8	8.1	4.3	10.5	3.1
Dicrotophos	0.006	81.8	85.7	7.4	1.2	16.7	5.1
Diethofencarb	0.004	79.3	90.2	5.5	5.5	17.3	3.0
Difenoconazole	0.005	89.0	88.0	4.9	5.6	14.4	13.0
Diflubenzuron	0.006	93.2	97.3	12.9	6.6	13.4	16.1
Dimefuron	0.003	87.5	93.2	8.4	10.3	9.3	4.2
Dimethachlor	0.003	81.7	95.7	17.2	7.4	4.3	2.7
Dimethenamid	0.003	94.3	93.1	7.2	5.0	4.5	4.7
Dimethoate	0.003	85.9	92.2	8.5	3.4	4.6	7.4
Dimethomorph	0.003	83.2	87.6	15.3	4.6	6.4	6.8

Dimoxystrobin	0.003	103.1	98.2	18.6	4.4	16.4	9.7
Diniconazole	0.006	85.2	91.8	5.8	4.3	13.6	6.9
Dinocap	0.003	76.4	85.7	6.5	5.4	16.8	3.3
Dioxacarb	0.003	81.3	93.9	6.2	2.9	9.9	9.7
Diphenamid	0.002	84.4	89.6	14.9	1.1	10.9	11.6
Diphenylamine	0.006	98.0	104.3	10.1	5.4	5.5	10.2
Disulfoton	0.003	108.2	101.3	9.0	5.6	0.7	1.9
Dithianon	0.007	99.9	96.5	10.5	3.5	16.8	6.3
Diuron	0.003	93.5	104.9	11.0	2.2	8.4	3.4
Dodine	0.002	91.2	92.2	9.4	3.7	16.1	9.2
Epoxiconazole	0.003	94.6	100.1	6.7	4.3	15.3	11.3
Etaconazol	0.003	80.9	93.0	15.2	2.8	10.6	2.3
Ethion	0.003	91.6	104.8	3.5	4.7	3.9	8.3
Ethirimol	0.002	87.2	87.5	12.7	5.7	8.1	13.0
Ethofumesate	0.006	87.2	96.3	5.1	5.0	19.8	6.6
Ethoprophos	0.003	91.3	105.9	5.9	5.4	17.1	13.7
Etofenprox	0.005	90.3	90.8	4.5	6.8	10.3	14.3
Etoxazole	0.003	84.9	83.4	10.7	5.8	6.3	16.2
Famoxadone	0.009	95.7	107.6	8.6	3.4	19.7	11.7
Fenamidone	0.003	84.2	86.9	14.0	2.3	17.6	4.4
Fenazaquin	0.003	81.7	88.2	2.7	3.9	5.5	18.0
Fenbuconazole	0.007	95.2	100.2	14.5	4.2	4.5	11.0
Fenhexamid	0.009	90.1	93.8	6.3	6.9	8.9	15.0
Fenobucarb	0.003	82.0	97.2	3.7	2.1	19.4	14.6
Fenothiocarb	0.002	101.6	114.7	8.2	7.6	5.4	6.3
Fenoxanil	0.007	93.8	102.5	10.5	8.8	7.8	4.1
Fenoxycarb	0.004	98.6	104.8	3.4	5.5	7.2	2.3
Fenpropathrin	0.009	99.6	92.1	5.3	3.3	11.1	4.4
Fenpropidin	0.003	83.6	87.0	11.3	7.1	8.9	11.4
Fenpropimorph	0.003	82.5	84.9	14.1	2.7	9.5	16.6
Fenpyrazamine	0.003	76.9	89.4	7.6	2.5	7.0	1.6
Fenpyroximate	0.005	83.9	81.7	11.3	2.2	10.7	5.0
Fenuron	0.002	95.4	89.6	7.7	7.0	8.5	5.3

Florasulam	0.009	85.6	91.8	19.1	3.4	11.7	4.3
Fluazinam	0.004	85.5	100.1	7.6	2.5	3.7	1.7
Flubendiamide	0.005	85.6	96.8	6.2	8.9	16.1	3.7
Fludioxonil	0.003	76.0	76.8	1.6	3.9	18.9	3.5
Flufenoxuron	0.007	82.6	83.0	6.4	4.2	7.7	6.1
Flumetsulam	0.002	99.4	93.6	10.4	8.2	7.9	3.5
Fluopyram	0.009	89.0	96.0	7.7	2.9	4.8	6.8
Flupyradifurone	0.005	83.0	92.0	4.7	3.8	13.1	3.9
Flutriafol	0.003	88.0	86.6	8.5	4.3	7.9	7.7
Fluxapyroxad	0.002	91.9	104.5	17.5	6.1	18.9	8.1
Formothion	0.004	87.5	85.8	8.7	4.3	9.2	3.6
Furathiocarb	0.002	82.6	90.9	16.3	3.7	18.8	9.2
Heptenophos	0.004	82.6	98.1	7.0	3.8	8.5	6.0
Hexaconazole	0.006	79.77	92.8	11.4	4.2	17.8	13.4
Hexaflumuron	0.006	88.6	75.7	6.1	3.2	10.3	7.5
Hexythiazox	0.006	87.5	81.8	5.6	6.0	7.5	18.3
Imazalil	0.004	84.0	84.0	3.9	6.1	5.5	5.9
Imazapyr	0.009	99.5	84.4	5.7	4.5	5.8	3.3
Imidacloprid	0.003	87.1	92.4	9.0	6.4	10.9	5.6
Indoxacarb	0.007	93.7	92.8	5.4	4.7	6.2	7.6
Ioxynil	0.008	87.4	84.3	5.3	5.3	11.2	4.3
Ipconazole	0.003	83.3	88.8	11.2	2.9	13.9	3.3
Isocarbamid	0.004	89.0	88.9	5.5	8.1	4.0	2.8
Isoprocarb	0.004	82.3	90.9	9.9	6.5	7.0	6.2
Isopropalin	0.005	86.6	87.6	8.5	5.6	7.2	3.4
Isopyrazam	0.003	80.9	95.9	8.6	2.4	17.4	6.0
Kresoxim-methyl	0.003	90.6	112.0	7.7	6.5	10.6	8.3
Lambda-cyhalothrin	0.009	95.4	102.1	10.4	4.0	10.6	4.0
Lenacil	0.004	87.3	102.7	15.5	7.8	11.3	16.8
Linuron	0.004	87.8	97.8	18.8	6.3	3.6	3.9
Lufenuron	0.006	98.2	94.3	8.5	5.1	5.1	8.5
Malaoxon	0.004	78.5	86.8	8.1	8.9	3.7	13.4
Malathion	0.003	86.6	100.6	13.4	3.2	12.3	13.0

Mecarbam	0.003	83.9	101.6	8.7	1.1	15.4	13.7
Metalaxyl	0.002	97.3	94.9	3.8	5.5	3.8	11.6
Metconazole	0.003	81.3	92.8	16.0	6.0	4.9	11.2
Methacrifos	0.006	84.0	96.2	15.6	7.1	13.5	9.3
Methamidophos	0.007	85.3	81.6	9.9	6.2	5.4	4.6
Methidathion	0.004	84.4	98.0	1.1	6.4	11.2	2.9
Methomyl	0.007	96.2	86.3	18.3	2.6	9.7	14.3
Methoxyfenozide	0.009	103.2	100.5	5.0	7.9	15.2	12.9
Metolachlor	0.004	84.1	98.4	7.9	5.8	17.0	8.0
Metosulam	0.003	91.8	92.0	2.2	5.1	17.9	10.7
Metoxuron	0.003	82.0	88.3	10.9	5.2	9.5	10.1
Metribuzin	0.009	87.1	90.7	6.8	4.7	14.3	11.4
Mevinphos	0.003	79.4	85.6	9.9	2.3	6.6	8.3
Monocrotophos	0.009	88.5	83.4	3.1	2.8	11.6	7.2
Monolinuron	0.002	81.4	93.2	10.7	5.8	12.9	10.0
Myclobutanil	0.004	77.7	92.2	11.0	3.1	14.5	15.9
Nicosulfuron	0.003	78.9	82.7	10.4	2.0	19.6	3.7
Nitenpyram	0.008	85.0	80.6	12.5	5.1	6.6	15.9
Novaluron	0.004	89.3	91.8	13.7	1.8	17.7	3.5
Nuarimol	0.003	77.1	87.9	4.8	7.2	5.9	4.3
Omethoate	0.008	83.0	97.4	11.1	3.8	6.0	4.1
Oxadixyl	0.009	98.7	101.4	6.1	4.1	15.9	8.5
Oxamyl	0.008	102.2	106.3	10.9	6.0	7.0	16.4
Penconazole	0.004	91.0	103.6	10.5	5.1	14.6	11.4
Pendimethalin	0.006	80.5	89.7	11.7	3.8	8.8	13.3
Phenmedipham	0.003	81.1	87.2	7.1	3.2	7.1	3.9
Phenthoate	0.004	97.7	115.3	4.7	8.0	4.5	11.3
Phosalone	0.006	95.3	104.2	4.8	6.2	14.5	5.7
Phosmet	0.003	84.2	100.1	2.7	3.9	19.7	10.1
Phosphamidon	0.003	77.8	81.2	5.3	4.3	7.3	12.2
Phoxim	0.002	92.8	106.2	11.1	1.7	12.1	8.4
Pirimicarb	0.003	74.3	84.1	5.8	7.8	5.1	6.6
Pirimiphos-ethyl	0.003	81.4	95.0	16.1	2.3	13.5	4.6

Pirimiphos-methyl	0.003	82.1	96.4	5.1	9.2	7.9	10.7
Primisulfuron	0.008	108.9	78.1	9.7	8.3	5.4	6.8
Prochloraz	0.005	82.6	95.8	3.3	7.0	15.6	8.9
Profenofos	0.003	89.9	103.5	3.1	6.1	8.7	17.0
Profoxydim	0.004	83.4	82.1	11.9	1.9	17.1	8.0
Prometryn	0.003	77.1	87.2	7.8	2.4	9.2	10.4
Propachlor	0.002	87.8	100.7	18.4	7.9	8.8	7.2
Propaquizafop	0.005	83.9	96.9	11.2	2.2	10.7	7.9
Propargite	0.004	81.5	95.4	2.6	6.6	12.1	10.6
Propham	0.009	95.2	89.7	8.2	3.4	19.0	7.5
Propiconazole	0.004	85.4	91.2	6.0	3.7	7.8	13.2
Propoxur	0.008	95.2	109.7	2.0	4.3	11.5	4.3
Pymetrozine	0.007	88.6	103.5	5.4	1.9	17.8	6.8
Pyraclostrobin	0.005	88.1	106.4	13.8	5.6	9.2	15.3
Pyrazophos	0.005	79.9	90.7	3.8	2.8	17.3	11.5
Pyridaben	0.004	82.6	87.0	3.0	5.4	16.2	6.8
Pyridaphenthion	0.006	80.9	95.6	3.9	4.9	13.6	11.1
Pyridate	0.005	79.1	87.2	15.6	1.9	17.8	7.8
Pyrifenox	0.002	86.5	100.6	14.7	7.7	5.6	11.2
Pyrimethanil	0.002	83.1	92.5	16.0	4.2	15.3	7.1
Pyriproxyfen	0.009	81.8	87.6	5.4	5.9	15.6	11.0
Rimsulfuron	0.004	82.6	82.4	6.5	5.1	8.2	9.5
Simazine	0.004	88.3	89.0	17.7	4.9	5.0	5.1
S-metalachlor	0.007	79.7	82.5	5.8	3.8	9.4	5.2
Spinetoram	0.007	100.3	97.1	8.4	2.9	15.9	1.4
Spinosad A	0.008	88.9	98.2	12.7	5.5	10.4	3.7
Spinosad D	0.004	92.3	108.6	6.6	6.5	5.0	5.5
Spirodiclofen	0.008	78.5	83.7	2.3	0.9	14.8	7.1
Spiromesifen	0.005	85.5	102.0	13.9	3.1	11.8	9.1
Spirotetramat	0.003	104.2	100.7	15.9	2.7	14.3	6.4
Spirotetramat enol glucoside	0.003	93.5	104.3	4.8	2.2	8.6	5.2
Spirotetramat mono hyrdoxy	0.005	101.6	103.5	5.4	3.0	7.2	5.0
Spirotetramat-enol	0.005	93.0	102.1	8.6	5.2	16.7	6.8

Spirotetramat-keto	0.003	101.5	103.4	5.5	2.0	10.3	10.6
Spiroxamine	0.002	78.4	83.3	6.1	4.9	6.0	8.2
Sulfosulfuron	0.004	86.0	91.7	13.2	3.3	8.2	3.8
Sulfotep	0.005	92.4	89.2	16.6	3.0	12.8	7.9
Sulfoxaflor	0.008	95.4	92.9	9.0	1.9	18.6	13.9
Tau-fluvalinate	0.007	95.7	103.2	5.9	3.7	16.2	4.1
Tebuconazole	0.006	88.9	95.2	4.3	5.3	11.8	12.0
Tebufenozide	0.004	79.8	92.3	7.2	9.1	13.2	14.6
Tebufenpyrad	0.003	78.9	91.3	3.0	4.5	6.2	10.7
Teflubenzuron	0.005	82.7	82.1	5.1	4.2	8.3	7.6
Terbufos	0.008	79.4	106.1	3.5	5.7	19.5	12.5
Terbutryn	0.002	77.1	87.2	13.0	3.0	18.9	10.1
Tetraconazole	0.005	86.4	96.5	10.4	7.5	13.8	12.4
Thiabendazole	0.002	97.9	85.8	7.3	1.9	9.8	2.5
Thiacloprid	0.002	79.5	90.7	4.4	6.0	12.5	6.9
Thiamethoxam	0.008	79.3	85.2	7.1	6.1	16.2	9.4
Thiobencarb	0.004	91.0	103.3	9.7	2.5	9.0	4.8
Thiodicarb	0.006	82.6	89.5	6.6	5.4	18.9	10.9
Thiophanate-methyl	0.007	109.5	101.8	9.4	7.0	6.7	4.8
Thiram	0.002	92.5	100.6	8.2	1.5	8.4	7.1
Tolylfluanid	0.005	109.8	105.7	15.3	5.0	12.8	14.2
Tralkoxydim	0.005	76.9	83.0	7.8	2.2	9.1	5.5
Triadimefon	0.003	79.5	94.2	12.4	4.6	10.2	6.8
Triadimenol	0.007	86.2	92.9	9.1	4.9	19.2	1.8
Triallate	0.009	99.7	91.7	13.5	2.6	7.9	4.2
Triasulfuron	0.005	100.5	101.4	12.8	5.0	10.9	5.6
Trichlorfon	0.007	85.7	87.5	12.1	5.8	7.2	4.8
Trifloxystrobin	0.002	80.2	94.2	1.4	5.7	18.6	11.4
Triflumizole	0.002	81.1	91.6	8.2	5.8	14.6	5.4
Triflumuron	0.007	89.7	91.1	8.7	5.4	15.2	4.6
Triticonazole	0.003	91.3	111.4	4.8	5.2	15.1	8.7
